# Treatment of Elektra prosthesis complications

**DOI:** 10.1186/1753-6561-9-S3-A89

**Published:** 2015-05-19

**Authors:** Pierre Jean Regnard

**Affiliations:** 1 SOS Mains Dijon, Point Medical, Dijon, 21000, France

## Introduction

We have been using the Elektra prosthesis for the treatment of osteoarthritis of the CMC joint of the thumb since 1997. After 760 implantations, we studied 689 cases to obtain sufficient follow-up in 596 women and 93 men. Of the 689 cases with sufficient follow up, only 661 cases were not deceased or had records that could be traced.

The results in this series are good in terms of mobility, strength and especially disappearance of pain as for other prostheses. Of note is that in this series spanning 15 years, all the cases were performed by a single surgeon.

## Materials and methods

197 cases with complications (dislocation, loosening) were found (28%), but 75 % occurred after severe trauma. For the isolated acute *dislocation* it was often possible to reduce without surgery, but in case of dislocation seen late, as in other joints, an open technique was necessary.

In case of *loosening*, specially loosening of the cup, we used a variety of techniques:

- Cancellous bone graft of the trapezium, and re-implantation of screwed cup in 33 cases with a good result in 80%.

- Cemented polyethylene cup in 150 patients, with a good result when the trapezium was still intact without fissure or fracture. But the wear of this implant is well known.

- Since 2013 we preferred to use the double mobility Moovis^®^ cup (11 cases), because the size of the implant is often sufficient to fill the cavity of the trapezium and thus it’s possible to give second chance to this prosthesis. The results were very good, except in one case where the trapezium was too destroyed. In this instance we performed interposition arthroplasty.

- Finally in 3 cases, it was not possible to re-implant the Elektra cup in the remaining trapezium because the fracture was from the distal edge of the bone, preventing the cup from “sitting” well.

## Discussion

In rare cases, we have seen allergy to nickel (4 patients), which is possible and in this complication early ablation of the prosthesis is required. One of the most difficult complications to manage is the pseudotumour or granuloma. This needs if possible, to resect the granuloma and to perform a cancellous bone graft, but if this lesion don’t disappear, complete removal of the prosthesis will be necessary.

## Conclusion

In conclusion, we think it’s possible to say that the Elektra is a reliable prosthesis, in view of the quality of the results excepting any trauma occurring. The possibility to correct each complication with specific treatment outlines the attraction of this prosthesis for the treatment of the CMC joint arthritis of the thumb.

